# The Dynamics of Oxidized LDL during Atherogenesis

**DOI:** 10.1155/2011/418313

**Published:** 2011-05-11

**Authors:** Hiroyuki Itabe, Takashi Obama, Rina Kato

**Affiliations:** Department of Biological Chemistry, Showa University School of Pharmaceutical Sciences, 1-5-8 Hatanodai, Shinagawa-ku, Tokyo 142-8555, Japan

## Abstract

Accumulating evidence indicates that oxidized low-density lipoprotein (OxLDL) is a useful marker for cardiovascular disease. The uptake of OxLDL by scavenger receptors leads to the accumulation of cholesterol within the foam cells of atherosclerotic lesions. OxLDL has many stimulatory effects on vascular cells, and the presence of OxLDL in circulating blood has been established. According to the classical hypothesis, OxLDL accumulates in the atherosclerotic lesions over a long duration, leading to advanced lesions. However, recent studies on time-course changes of OxLDL *in vivo* raised a possibility that OxLDL can be transferred between the lesions and the circulation. In this paper, the *in vivo* dynamics of OxLDL are discussed.

## 1. The Background of OxLDL Hypothesis

In the last three decades, a large volume of studies have established that oxidized low-density lipoprotein (OxLDL) is a useful marker for cardiovascular diseases (CVDs) (see [1–6]). The measurement of OxLDL correlates with the presence of CVDs and indicates that OxLDL is a potential prognostic marker for future health events. OxLDL is known to stimulate macrophages to induce foam cell formation and inflammatory responses. Although the pathological aspects of OxLDL have been well studied, the formation, distribution, and overall fate of OxLDL *in vivo* remain unclear.

The LDL receptor discovered by Goldstein and Brown has central roles in the systemic and cellular metabolism of cholesterol [7]. Cholesterol accumulates in atherosclerotic lesions even in patients with familial hypercholesterolemia having genetically impaired LDL receptor. This observation raised the question of how lipid-laden foam cells are formed without LDL receptor. They also suggested the importance of recognition of modified LDL by specific receptors. By using acetylated LDL as the model ligand, they demonstrated the presence of new receptors that facilitate foam cell formation, which were called scavenger receptors [8]. Scavenger receptor type A (SR-A), the first scavenger receptor to be identified, is responsible for the recognition and uptake of acetylated LDL and OxLDL [9, 10], but SR-A is not the sole receptor for modified LDL. More than 10 scavenger receptors have been identified so far [11–13].

In 1984, Steinbrecher and his colleagues proposed that oxidative modification promotes foam cell formation from macrophages and that the oxidative modification of LDL can be achieved through the interactions of LDL with endothelial cells *in vitro* [14]. OxLDL induces a number of proatherosclerotic effects, including endothelial activation and smooth muscle proliferation [14–19]. Berliner et al. reported that the oxidative modification of LDL through coculture with endothelial cells had stimulatory effects on many types of cells and that these effects are due to the oxidized phospholipids generated in OxLDL [15]. The accumulation of OxLDL in the foam cells in atherosclerotic lesions from human coronary arteries, carotid arteries, and aortas of Watanabe heritable hypercholesterolemic (WHHL) rabbits has been demonstrated by immunohistochemical analysis using anti-OxLDL monoclonal antibodies [20–23].

Since the clearance of OxLDL from the circulation is so efficient [24], OxLDL was believed initially to be absent in blood. Anti-OxLDL monoclonal antibodies were applied to sensitive ELISA procedures to measure very small amounts of OxLDL in circulating blood [25–28]. Several research groups, including ours, have demonstrated that the plasma OxLDL level in patients with CVDs is significantly higher than the level measured in healthy subjects [21, 29–31]. The ELISA procedures introduced by these groups were different so that the values of the OxLDL measurements cannot be directly compared; however, they all reported an increase in OxLDL levels in patients with acute myocardial infarction (AMI), cerebral infarction, or those receiving hemodialysis. These observations strongly suggest that circulating OxLDL is involved in atherosclerosis. The details of the ELISA procedures used and the clinical observations have been summarized previously [4].

It is still unclear how and when LDL is being oxidized. It has been long believed that LDL is modified in vessel wall and subsequently accumulates in the tissue. However, Tsimikas et al. showed that plasma OxLDL increases concomitant with the regression of atherosclerotic lesions [32], suggesting that OxLDL can be transferred between vessel wall and the circulation.

 Recently, the importance of the plasma OxLDL level as a plausible predictive marker for secondary prevention has been discussed [33–36]. To understand the role of OxLDL in atherogenesis, it is important to clarify the behavior of OxLDL *in vivo *during atherogenesis, particularly how OxLDL is formed, how it moves around tissues, and how it is metabolized. In this paper, we briefly review recent advances regarding dynamics and kinetics of OxLDL *in vivo* and suggest several possibilities for future research.

## 2. Temporal Changes in Plasma OxLDL Levels

We and others started to measure human plasma OxLDL levels using sandwich ELISA procedures [25–27]. The combination of two antibodies, one for oxidized phosphatidylcholines (OxPC) and the other for human apolipoprotein B (apoB), enables accurate and efficient detection of OxLDL; as low as 1 ng/mL of OxLDL can be measured. In the last 15 years, many clinical studies were conducted using these techniques. OxLDL levels increase in the plasma of patients with several pathological conditions, including CVDs [21, 29, 30, 33], cerebral infarction [37], and hemodialysis for the treatment of chronic renal failure [25, 28, 38]. This evidence strongly suggests the involvement of *in vivo* OxLDL in atherosclerosis and indicates that OxLDL levels may increase in patients with symptoms of atherosclerosis. In addition, recent studies demonstrated that temporal rises and falls in plasma OxLDL levels occur under certain conditions. 

Acute myocardial infarction (AMI) is caused by total occlusion of a coronary artery following the rupture of an atherosclerotic plaque and acute thrombosis. Ehara et al. reported that the plasma OxLDL level in patients with AMI increased by approximately 3.5 fold than in control subjects [21]. However, this increase in the plasma OxLDL level appeared to be a temporal change during followup studies on the AMI patients. The increased plasma OxLDL levels dropped to near the normal range by the time the subjects were discharged from the hospital [33]. Similar behavior of plasma OxLDL level was observed in patients with cerebral infarction. The plasma OxLDL levels of these patients increased for the first few days following onset of the disease, then returned to normal range within 30 days [37]. Similar temporal changes in plasma OxLDL levels have been reported in patients with acute infarction of the coronary arteries and after percutaneous transluminal coronary angioplasty (PTCA) treatment [31, 39, 40]. AMI is induced by spontaneous plaque rupture, and the plaque can be further damaged by following PTCA treatment with a balloon catheter and stent. Increased plasma OxLDL levels after these acute events are partially due to the release of OxLDL from the atherosclerotic plaques upon rupture [41]. 

**Figure 1:**
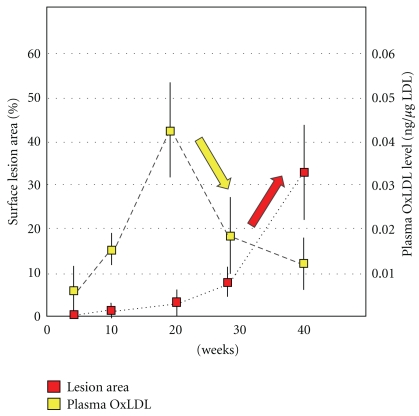
Temporal changes in plasma OxLDL levels and atherogenesis in apoE-KO mice. Male apoE-KO mice were maintained on normal diet up to 40 weeks. The atherosclerotic lesion on the aortic surface increased sharply after 20 weeks. Plasma OxLDL levels increased at 20 weeks just before the lesions began growing. The plasma OxLDL levels seem to decrease concomitantly with an increase in the size of the lesion (cited from [41] with modification).

The time-course behavior of OxLDL during the early stages of atherogenesis was investigated. To utilize apolipoprotein E knockout (apoE-KO) mice, a modified sandwich ELISA procedure was used to determine the murine circulating OxLDL level [42]. ApoE-KO mice develop atherosclerotic lesions even when fed normal diet. The lesion area was less than 1% of the whole aortic surface at 10 weeks, but it gradually increased after 20 weeks, and it grew up to 32% by 40 weeks of age (Figure 1). The OxLDL level was 0.006 ng/micro g LDL at 6 weeks, but it was elevated to 0.042 ng/micro g LDL at 20 weeks and then decreased by 60% to 0.018 ng/micro g LDL at 28 weeks. This temporal rise in OxLDL occurred before the size expansion of atherosclerotic lesions in the aorta. This observation suggests that the OxLDL is generated during atherogenesis in apoE-KO mice *in vivo.* Reduction in the plasma OxLDL level coincided with the increase in the development of the lesion and accumulation of OxLDL in the atherosclerotic intima. One possible explanation is that plasma OxLDL can transfer into intimal lesions.

Tsimikas et al. studied plasma OxLDL levels (OxPC/apoB ratios) during the regression of atherosclerosis in adult cynomolgus monkeys and New Zealand white rabbits using anti-OxPC and anti-apoB monoclonal antibodies [32]. They showed that OxLDL levels correlated inversely with changes in the size of lesions. Plasma OxLDL levels decreased during the progression of lesions under a high-fat diet and increased during the regression of these lesions after the diet was changed to normal. This observation also suggests that OxLDL can be transferred between the intimal regions and the circulation.

**Figure 2:**
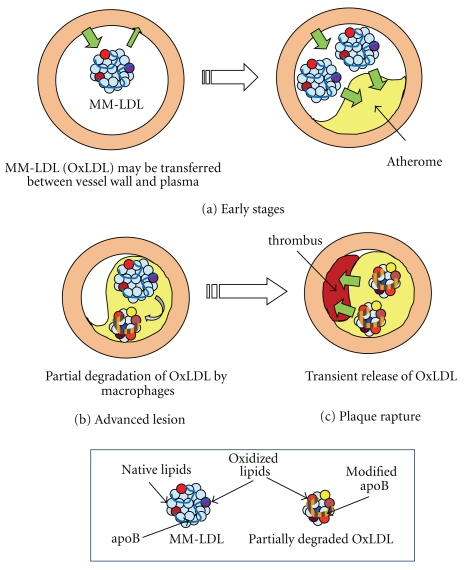
Possible* in vivo* behavior of OxLDL. (a) The temporal rise and fall of plasma OxLDL levels suggest that MM-LDL (OxLDL) may be transferred between the vessel wall tissues and circulation in the early stages of atherogenesis. In this stage, circulating OxLDL is likely to be MM-LDL, since heavily oxidized LDL is very rapidly cleared from the circulation. The tissues of the vessel wall could be the site of LDL oxidation, but further study is needed to examine whether oxidation proceeds in apparently healthy vessel walls during the very early stages. When the plasma OxLDL level decreases atherosclerotic lesions appear to develop. (b) In advanced stages, many macrophages and foam cells are found in the atherosclerotic lesions. MM-LDL could be further modified to form OxLDL in the lesions. OxLDL is taken up by macrophages, and processed in the lysosomes. Some of the OxLDL is completely degraded, and a part of OxLDL is relatively resistant to proteolytic processing. Partially degraded OxLDL particles are observed in the lesions. (c) Upon plaque rupture, or when an atherosclerotic plaque is injured by PTCA treatment, OxLDL and partially degraded OxLDL are rapidly released from the lesions into the circulation, which causes a temporal rise of the plasma OxLDL level.

Atherosclerotic lesions can regress under certain conditions, such as a low-fat diet regimen or treatment with a lipid-lowering drug. In human studies, increases in plasma OxLDL levels was reported in healthy volunteers fed a low-fat diet [43]. After 37 healthy women took low-fat low-vegetable diet for 5 weeks, OxPC/apoB ratio increased by 27%, while total cholesterol did not change. In MIRACL study, patients with unstable angina pectoris or AMI were treated with atorvastatin (80 mg/day) for 16 weeks. Such treatment decreased total cholesterol and total apoB but increased OxLDL levels by 9.5% [44]. These observations suggest the possibility that OxLDL could translocate between atherosclerotic lesions and circulation. The rupture of a plaque could cause the rapid release of OxLDL into the circulation, but this may not be the only way to transfer OxLDL from the lesions into circulation. According to these recent studies, without tissue damage, OxLDL may possibly be equilibrated between the plasma and tissues (Figure 2). 

In addition, trapping circulating OxLDL by injecting anti-OxLDL antibodies or soluble form of the scavenger receptor LOX-1 induced regression of atherosclerotic lesions [45, 46]. Although that lipoprotein metabolism in rodents may have some difference from that of humans, it is nice if the regression therapies of atherosclerosis become available.

## 3. The Site of OxLDL Formation

 One of the major issues regarding OxLDL is the site of OxLDL formation *in vivo*. The most common way to prepare OxLDL is to incubate isolated LDL fractions with micromolar concentrations of copper sulfate for 3–24 hours. The copper ion induces lipid peroxidation chain reactions, and, subsequently, the chemical modification of the apoB protein side chains with reactive lipid peroxidation products, such as 4-hydroxynonenal (4-HNE), acrolein, and malondialdehyde (MDA). The presence of adducts of these oxidized products on apoB protein has also been immunologically confirmed in atherosclerotic lesions [47–49]. Although concentrations of metal ions are typically low in the plasma, including copper ion, sufficient amounts of reducing compounds and proteins exist in the plasma that can protect LDL from oxidation [47]. However, whether copper ion-induced LDL oxidation occurs under physiological conditions remains unclear [41].

**Figure 3:**
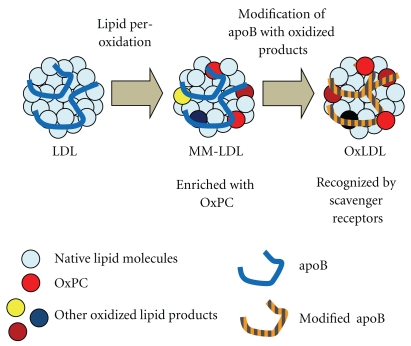
Difference between OxLDL and MM-LDL. LDL is thought to be modified in a stepwise manner during the generation of OxLDL. In the initial phase of modification, the lipid components (sky blue circle) react with oxidation reagents, resulting in radical chain reactions that produce many types of lipid oxidation products (red, brown, yellow, or dark blue circle). Then, the lipid oxidation products react with the apoB protein (blue line) to generate adducts and cross-links. Radicals can attack the apoB protein directly, resulting in oxidative changes of amino acid side chains and the cleavage of peptide bonds (orange-grey line). MM-LDL may contain lipid oxidation products without extensive protein modification, because it binds to LDL receptor rather than scavenger receptors. As modification on the apoB protein proceeds, its mobility in the agarose gel electrophoresis changes greatly, and it loses the affinity to LDL receptor, and, in turn, it becomes a ligand of scavenger receptors.

 Cell culture-dependent modification of LDL has been used to prepare “minimally modified LDL” (MM-LDL), which is one form of oxidatively modified LDL [14, 15]. The chemical modification of MM-LDL is moderate judging by increases in thiobarbituric acid-reactive substances (TBARSs) and mobility in agarose gel electrophoresis, so that MM-LDL binds to LDL receptor rather than scavenger receptors. However, MM-LDL showed strong inflammatory effects on the cells in vessel wall tissues [15–17, 50]. MM-LDL contains substantial amounts of OxPC, and OxPC is believed to be partially responsible for these biological effects [51–53]. Sandwich ELISA system that uses the anti-OxPC monoclonal antibody binds as competently to MM-LDL as to copper-induced OxLDL [54]. Circulating OxLDL may be qualitatively similar to MM-LDL, since OxLDL can escape clearance system by the scavenger receptors and is immunologically positive to measurement (Figure 3).

 Heinecke et al. reported that chlorotyrosine and nitrotyrosine, which are generated by the myeloperoxidase-(MPO-) dependent modification of proteins, accumulate in atherosclerotic lesions and in circulating LDL [55, 56]. MPO, an enzyme that is secreted from macrophages and neutrophils, is involved in antibacterial host defense through the generation of reactive oxygen species. MPO could oxidize LDL particles, and the resulting OxLDL is taken up by macrophages to form foam cells [57]. Since MPO is able to modify LDL even in the presence of plasma *in vitro*, MPO secreted by the macrophages and/or neutrophils of atherosclerotic lesions could induce OxLDL formation. According to this hypothesis, OxLDL is formed in inflammatory tissues rather than in the blood.

 Wen and Leake reported an interesting possibility to form OxLDL [58]. They incubated macrophages with aggregated LDL to form foam cells. When the foam cells were cultured for 7 days, 7-ketocholesterol and lipofuscin-like fluorescent materials were formed in the intracellular vesicles of the foam cells. Since oxidative changes were inhibited by antioxidants and by the lysosomotropic agent chloroquine, they proposed that LDL can be oxidized within the lysosomes of macrophages. 

 We recently found the presence of OxLDL in human gingival crevicular fluid (GCF). Periodontitis, a major cause of tooth loss in many countries, is a chronic infectious disease that leads to the destruction of the connective tissue attachments and alveolar bone. Recent studies have suggested that a correlation between atherosclerosis and periodontal disease exists [59, 60]. GCF is a plasma exudate from the microcirculation of gingival tissue, which is located in the tiny space between the gingiva and tooth, and GCF secretion increases in patients with periodontal disease [61]. GCF contains several plasma proteins, including cytokines, hormones, and protective proteins to bacterial infection [62–64], but the protein profile of GCF is different from that of peripheral plasma. Using paper points, up to 1 *μ*L of GCF samples can be easily collected without damaging the tissue, and the lipoprotein and cytokine concentrations can be investigated. We found that LDL was present in GCF. In addition, OxLDL was present in GCF samples collected from healthy subjects. Surprisingly, the OxLDL level in the GCF samples was 14-fold higher than in the plasma OxLDL levels obtained from the same subjects [65]. The study on the GCF from the periodontal lesions is ongoing. We demonstrated that OxLDL induces IL-8 production in Ca9-22 human gingival epithelial cells in culture, suggesting that OxLDL in GCF could function as an inflammatory stimulant [66]. If OxLDL in GCF is simply transferred from circulation, the ratio of OxLDL to LDL (same as OxLDL levels) should be the same as that in plasma. These results suggest that the OxLDL in GCF could be formed in periodontal pockets or local gingival tissues rather than simply being transferred from the plasma. It is an interesting view point that OxLDL could be generated in extra-arterial tissues.

## 4. The Possible Fate of OxLDL

Scavenger receptors are a set of receptors that bind to OxLDL but not to native LDL particles. Macrophages and related cells, such as Kupffer cells, express some of the scavenger receptors, such as SR-A and CD36, but it is now apparent that other types of cells such as endothelial cells also possess other scavenger receptors [11–13]. Currently, scavenger receptors are considered to be a set of pattern recognition receptors that are expressed by the innate immune-defense system against various pathogens [67, 68]. OxLDL and/or the oxidized phospholipid components of OxLDL are also recognized by Toll-like receptors 2 and 4 [69, 70], indicating an overlap of the modification structures on OxLDL and nonself patterns recognized by defense system. Scavenger receptors recognize OxLDL particles, because their modified structures are similar to pathogen-related epitopes thereafter, OxLDL is removed from the milieu by endocytosis. This process is a part of self-defense responses, however, it may cause accumulation of massive amount of lipids inside macrophages.

*In vivo* evidence of OxLDL accumulation in foam cells was obtained by immunohistochemical analysis [20–23]. Using an anti-OxPC monoclonal antibody, OxPC accumulation was clearly observed in the cytoplasmic space of foam-cell macrophages in the aortic lesions of WHHL rabbits [20] and atherosclerotic lesions in human coronary arteries obtained from an autopsy specimen [21]. OxPC and apoB strictly colocalized in the foamy macrophages of the lipid-rich core of atherosclerotic lesions.

ApoB is a large protein with a molecular weight of approximately 500 kDa. During oxidative modification, aggregation of LDL with conjugated apoB is formed. However, partially degraded apoB fragments were found in atherosclerotic lesions. On examining the LDL fraction extracted from lesions of the human carotid artery by Western blotting with anti-apoB monoclonal antibody, the size of apoB proteins in the LDL extract was 100–200 kDa [22] (Figure 2(b)). Partial degradation of OxLDL in macrophages was observed using macrophages *in vitro*, whereas acetylated LDL is completely degraded in the macrophages [71, 72]. Cross-linking of apoB protein formed in OxLDL may contribute to this resistance to proteolysis. OxLDL is possibly processed by lysosomal enzymes in foam cells. For future research, investigating whether plasma OxLDL is partially degraded in patients with AMI during the acute phase would be interesting. Since the temporal rise and fall of plasma OxLDL levels after plaque rupture are believed to be caused by release from atherosclerotic lesions, such information would be an important clue for elucidating the origin of OxLDL in the circulation. 

 Another possibility that should be considered is whether Lp(a) is a circulating OxLDL. A prospective clinical study that examined the relationship between OxLDL and future cardiovascular events showed a close overlap of plasma OxLDL level and Lp(a) concentration [73]. The human LDL fractions were further separated into subfractions by density, and OxPC was found to be enriched with the subfractions that contained Lp(a) [74]. Lp(a) is a unique LDL particle in which apoB protein binds to another protein containing repeated kringle domains, apolipoprotein(a), through a disulfide bond. Apolipoprotein(a) has some similarities with plasminogen, but the physiological function of Lp(a) is largely unknown.

The liver is the major organ responsible for OxLDL metabolism. When radiolabeled OxLDL is injected into rats via the femoral vain, most of the radioactivity is cleared from circulation within 3 minutes, and more than 70% of radioactivity goes to the liver [24]. OxLDL has been documented to accumulate in atherosclerotic lesions in the aorta and coronary arteries of patients with CVDs; however, the possible roles and pathological changes that occur in the liver of patients with CVDs have not been studied in depth. The proteolytic capabilities of liver cells may possibly be different from those of the macrophages in the vessel wall. 

**Figure 4:**
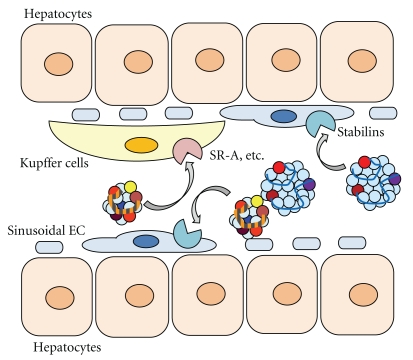
Stabilins, novel endothelial scavenger receptors, could have a role in the clearance of OxLDL in the liver and from circulation. Heavily oxidized LDL is taken up by Kupffer cells, whereas sinusoidal endothelial cells can take up both mildly and highly oxidized form of LDL. The novel scavenger receptors stabilin-1 and -2, which can bind to both types of OxLDL, could be involved in the process of clearing OxLDL from the circulation in the liver.

Kupffer cells, the macrophage-like cells of the liver, express scavenger receptors, including SR-A. However, it was surprising that the clearance rate of OxLDL from circulation in SR-A knockout mice was similar to that in normal mice [75]. This suggests that there might be alternative receptors or systems for the uptake of OxLDL in liver cells. Recently, Li et al. reported that both Kupffer cells and sinusoidal endothelial cells in the liver endocytose OxLDL and MM-LDL, and this process is mediated by the novel scavenger receptor stabilin-2 [76]. Stabilins, specifically stabilin-1 and stabilin-2, were initially found as receptors for hyarulonan [77, 78]; however, they were identical to FEEL-1 and FEEL-2, which are reported to be endothelial scavenger receptors [79]. These two stabilins are expressed in the sinusoidal endothelial cells of the liver but show different ligand specificities. Stabilin-1 binds to the both mildly and highly oxidized forms of LDL, while stablin-2 selectively binds to the highly oxidized LDL. When nonparenchymal cells, which contain both sinusoidal endothelial cells and Kupffer cells, were incubated with these two forms of OxLDL, the uptake of mildly oxidized form of LDL was observed only in endothelial cells, whereas both cells were capable of taking up highly oxidized LDL. The co-localization of OxLDL and stabilins has been demonstrated by immunoelectronmicrography. These observations suggest that endothelial cells might have a significant role in the metabolism of circulating modified LDL. If MM-LDL represents the circulating form of OxLDL, endothelial cells may be an important site of accumulation, metabolism, and further modification of MM-LDL (Figure 4).

 Certain scavenger receptors, such as SR-A, CD36, and LOX-1, have been extensively studied to determine their relationship to foam cell formation and atherogenesis. However, other receptors for OxLDL may possess different tissue distributions and functions. Among scavenger receptors, SREC and CL-P1 have been reported to be selectively expressed in endothelial cells [80, 81], but the physiological functions of these special receptors have not been clarified. To elucidate the clearance of OxLDL, studies on the contribution of endothelial cells and the specialized receptors are required.

## 5. Conclusion

 Recent studies have suggested the plasma OxLDL concentrations may change under prepathological and postpathological conditions. OxLDL may be transferred between tissues and plasma and does not merely accumulate in the lesions but is equilibrated between the tissues and circulation. OxLDL can be formed in various sites in addition to the tissue of the vessel wall. The liver is the major organ for the clearance of OxLDL from circulation. However, many unknowns remain to be elucidated regarding the metabolic fate of OxLDL in the liver. A recent study pointed out that stabilins may have an important role in the recognition and clearance of OxLDL and MM-LDL from circulation. The receptors working in the liver may be different from those of cells in vessel wall tissues. Further studies are needed to understand the *in vivo* behavior of OxLDL and elucidate the contributions of OxLDL and oxidative stress to the mechanism of atherogenesis.
